# Marine-derived fungus *Aspergillus* cf. *tubingensis* LAMAI 31: a new genetic resource for xylanase production

**DOI:** 10.1186/s13568-016-0194-z

**Published:** 2016-03-24

**Authors:** Juliana A. dos Santos, Juliana M. F. Vieira, Alexandre Videira, Lucas A. Meirelles, André Rodrigues, Marta H. Taniwaki, Lara D. Sette

**Affiliations:** Departamento de Bioquímica e Microbiologia, Universidade Estadual Paulista Júlio de Mesquita Filho—UNESP, 24A, 1515, Rio Claro, SP 13506-900 Brazil; Instituto de Tecnologia de Alimentos—ITAL, Campinas, SP Brazil

**Keywords:** Xylanase, Marine-derived fungi, Marine biotechnology, Experimental design

## Abstract

Marine-derived fungi have been reported as relevant producers of enzymes, which can have different properties in comparison with their terrestrial counterparts. The aim of the present study was to select from a collection of 493 marine-derived fungi the best producer of xylanase in order to evaluate the enzymatic production under different conditions. A total of 112 isolates produced xylanase in solid medium containing xylan as the carbon source, with 31 of them able to produce at least 10 U/mL of the enzyme. The best production (49.41 U/mL) was achieved by the strain LAMAI 31, identified as *Aspergillus* cf. *tubingensis.* After confirming the lack of pathogenicity (absence of ochratoxin A and fumonisin B_2_ production) this fungus was submitted to the experimental design in order to evaluate the effect of different variables on the enzymatic production, with the aim of optimizing culture conditions. Three experimental designs (two Plackett–Burman and one factorial fractional) were applied. The best condition for the enzymatic production was defined, resulting in an increase of 12.7 times in comparison with the initial production during the screening experiments. In the validation assay, the peak of xylanase production (561.59 U/mL) was obtained after 96 h of incubation, being the best specific activity achieved after 72 h of incubation. Xylanase from *A.* cf. *tubingensis* LAMAI 31 had optimum pH and temperature at 5.0 and 55 °C, respectively, and was shown to be stable at a range of 40–50 °C, and in pH from 3.6 to 7.0. Results from the present work indicate that *A.* cf. *tubingensis* LAMAI 31 can be considered as a new genetic resource for xylanase production.

**Electronic supplementary material:**

The online version of this article (doi:10.1186/s13568-016-0194-z) contains supplementary material, which is available to authorized users.

## Introduction

Xylan is the main component of hemicelluloses, which is the second most abundant polysaccharide in nature, accounting for about one-third of all renewable organic carbon on earth (Zhou et al. 2009) and comprising approximately 25 to 30 % of the vascular plant masses (Gomez et al. 2008). Xylan biodegradation requires a set of enzymes (xylanases), which hydrolyze the main chain of xylan to oligosaccharides, subsequently degraded to xylose (Chávez et al. 2006). Xylanases have great biotechnological potential in the development of environmental friendly technologies in different sectors, including the paper and pulp and the feed and food industries. These enzymes are also applied in the generation of liquid fuels and chemicals from lignocellulose (Juturu and Wu 2012). In nature, there are numerous microorganisms that efficiently degrade xylan, being filamentous fungi one of the best degraders due to their great capability of secreting a wide range of xylan-degrading enzymes (Collins et al. 2005).

Filamentous fungi are particularly interesting as plant cell wall-degrading enzyme producers, since they secrete these enzymes directly into the medium, liberating energy and nutrients from plant biopolymers (Aro et al. 2005). According to Polizeli et al. (2005) this physiological characteristic is especially interesting because it eliminates the need for cell disruption in an industrial process and the levels of fungal extracellular enzymes generally are higher than those found in yeasts and bacteria.

The main activity of fungi in marine environments is associated with the decomposition of organic matter. Bonugli-Santos et al. (2015) suggested that hydrolytic and/or oxidative enzymes (including xylanases) are produced by several species of marine fungi. However, despite this potential, xylanases especially from marine filamentous fungi have been poorly studied.

The compounds produced by fungi from marine origin may exhibit structural differences when compared with that produced by fungi isolated from other environments, due to the adaptation to marine environment conditions (Bugni and Ireland 2004; Saleem et al. 2007). Microorganisms from marine environments are able to produce enzymes with great flexibility in relation to pH and temperature, while maintaining high specific activity (Trincone 2010, 2011; Bonugli-Santos et al. 2015). Due to the properties of enzymes produced by fungi from marine origin, such as thermal stability, tolerance to high salt concentrations and pressure, they are able to catalyze chemical reactions that are not catalyzed by the enzymes produced by their terrestrial counterparts (Saleem et al. 2007; Zhang and Kim 2010).

In this sense, the main objectives of the present study were to screen a collection of marine-derived fungi to select the best xylanase producer, to optimize the culture conditions for enhancing the enzymatic activity produced by the selected fungus, and to evaluate the characteristics of crude enzyme obtained under optimized conditions.

## Materials and methods

### Microorganisms

The filamentous fungi (n = 493 isolates) from the north coast of São Paulo State (Brazil) were isolated by Menezes et al. (2010). The selected fungus *A.* cf *tubingensis* was deposited at the Brazilian Collection of Microorganisms from Environment and Industry (CBMAI/UNICAMP) and at the Microbial Resource Center (CRM-UNESP) under the number CBMAI 1232 and CRM 523, respectively.

Fungi were isolated from samples of marine macroorganisms, including the sponges *Amphimedon viridis, Axinella corrugata, Dragmacidon reticulatum, Geodia corticostylifera, Mycale laxissima,* and *Mycale angulosa*; the ascidians *Didemnum ligulum* and *Didemnum* sp.; and the algae *Sargassum* sp. Samples were collected in January 2007 from the following beaches: Praia Guaecá (23°49′S; 45°25′W), Ilha Toque–Toque (23°51′S; 45°31′W), and Ilhota da Prainha (23°51′S; 45°24′W), São Sebastião region, São Paulo State, Brazil, at depths between 5 and 10 m (Menezes et al. 2010). In addition, fungi were isolated from the sponge *Dragmacidon reticulatum* collected from 29 November to 06 December 2008 at the following beaches: Saco do Poço (23°45′S; 45°15′W) on the east region of Ilha Bela and Costa do Aquário (23°47′S; 45°09′W) on Ilha de Búzios São Paulo State coastline, at depths between 5 and 10 m (Passarini et al. 2013).

### Screening of xylanase activity

Marine-derived fungi were inoculated separately in tubes containing 1 mL of YNB medium (yeast nitrogen base) with xylan as the sole carbon source. The cultures were incubated at 28 °C and 120 rpm for 7 days. After this period, the enzymatic broth (supernatant) was obtained by centrifugation (15 min at 3000 rpm). Aliquot of 25 μL of the supernatant was placed in sterilized straws arranged into Petri dishes containing agar-xylan medium. The plates were incubated for 48 h. After incubation time, the straws were removed and the culture medium was colored with lugol. The presence of a degradation halo in the culture medium was indicative of the presence of enzymes (Costa 2014).

### Culture conditions for enzymatic production

Fungal strains were cultivated in 2 % (w/v) malt extract agar (MA2) for 7 days at 28 °C. After this period, three fungal plugs (0.5 cm in diameter) taken from the edge of the colony were transferred to 200 mL Erlenmeyer flasks containing 50 mL of the Mandels and Sternbergs medium (MS), containing in g/L: 1.0 peptone, 1.4 (NH_4_)_2_SO_4_, 2.0 KH_2_PO_4_, 0.3 urea, 0.3 CaCl_2_, 0.3 MgSO_4_.7H_2_O, 0.005 FeSO_4_.7H_2_O, 0.001 MnSO4.H2O, 0.001 ZnSO_4_.7H_2_O, 0.002 CoCl_2_, pH 6.0, with 10.0 birchwood xylan (Sigma-Aldrich) as the carbon source. Assays were incubated for 7 days at 140 rpm and 28 °C. Cultures were harvested by centrifugation at 12,074×*g* for 30 min and the supernatant was used for the enzymatic quantification.

### Assay of xylanase activity

Enzymatic activity was measured spectrophotometrically (Biochrom, Libra S60) in triplicate. Xylanase activity was quantified by determining the amount of reducing sugar released from xylan derived from birchwood according to Bailey et al. (1992). The xylanase activity assay was performed by adding 20 μl of enzymatic broth into 50 mM citrate buffer (pH 5.0) with 1 % (w/v) birchwood xylan (Sigma) at 40 °C for 5 min. The generated reducing sugar was measured by using 3.5 dinitrosalicylic acid (DNS) (Miller 1959). One unit of enzyme activity was defined as the amount of enzyme required to release 1 μmol of product equivalent per min in assay conditions.

### Identification of isolate LAMAI 31

Genomic DNA was extracted from mycelium grown on PDA for 7 days at 28 °C following a modified version of the CTAB method (Möller et al. 1992; Gerardo et al. 2004), and amplified using the primer pair F-Bt2a (5′GGTAACCAAATCGGTGCTGCTTTC3′) and R-Bt2b (5′GGTAACCAAATCGGTGCTGCTTTC3′) described in Glass and Donaldson (1995). Conditions used for amplification were 3 min of denaturation at 94 °C, 35 cycles of 1 min at 94 °C, 58 °C for 30 s, and 2 min of extension at 72 °C. Amplicons were cleaned up with Kit Wizard SV Gel (Promega) following the manufacturer’s protocol. Cycle sequencing reactions were carried using BigDye Terminator v. 3.1 Cycle Sequencing Kit (Life Technologies) following the manufacturer’s instructions. Both forward and reverse sequences were generated using ABI3130 Genetic Analyser (Life Technologies) using the same primer pair. Contigs were assembled in Bioedit v. 7.2.5 (Hall 1999) and queried using the NCBI-GenBank to find the closest known sequences.

An alignment was built using 17 beta tubulin sequences retrieved from the study of Jurjevic et al. (2012). Thus the total number of sequences used in the alignment was 18. All sequences were aligned in MAFFT v.7 (Katoh and Standley 2013) and after final trimming this consisted in 604 base pairs. We carried out phylogenetic analysis in MEGA v. 6 (Tamura et al. 2013). The Kimura 2-parameter model (Kimura 1980) was used to estimate evolutionary distance for the Maximum Likelihood analysis. Branch support was calculated by bootstrap with 1000 replicate runs. A sequence of *Aspergillus violaceofuscus* was used as the outgroup. Beta tubulin nucleotide sequence from *A*. cf. *tubingensis* LAMAI 31 was deposited at GenBank under the accession number KT935448.

### Evaluation of toxic properties

#### Fumonisin B_2_ (FB_2_)

The strain LAMAI 31 was grown on Czapek yeast extract 20 % sucrose (CY20S) at 25 °C for 7 days, according to the method reported by Frisvad et al. (2007). After incubation, 5 plugs from the central part of the colony were cut and placed in vials. An amount of 1 mL of methanol was added and stirred in a vortex for 3 min. To clean up the extract a syringe was used to carry out two filtrations with Millex (0.45 and 0.22 µm). An aliquot of 100 µl of the filtrate was collected and transferred to a vial adding 100 µl of methanol. The vial was stirred in a vortex and 55 µl of the extract was transferred to a vial adding 55 µl of o-phthalaldehyde (OPA) reagent for fumonisin derivatization (Visconti et al. 2001).

Detection of FB_2_ was performed using high performance liquid chromatography (HPLC) following the conditions recommended by Visconti et al. (2001). Chromatography from Shimadzu LC-10VP (Shimadzu, Japan), with a fluorescence detector at 335 nm excitation and 440 nm emission was used. The mobile phase used acetonitrile: water: acetic acid (51:47:02 v/v/v). The mobile phase was filtered through a 0.45 mM membrane. The flow of the mobile phase was set to 1.0 ml/min. The oven temperature was 40 °C. An FB_2_ standard (Sigma Chemical Co., St Louis, USA) was used for the comparison.

#### Ochratoxin A

The isolate LAMAI 31 was grown on Yeast extract 15 % sucrose agar (YESA) at 25 °C for 7 days and evaluated for the production of OTA by the agar plug technique, which tests small samples from Petri dishes by thin layer chromatography (TLC) (Filtenborg et al. 1983). The TLC plates were developed in toluene/ethyl acetate/formic acid (5:4:1) and visualized under UV light at 365 nm. An OTA standard (Sigma Chemical Co., St Louis, USA) and *A. carbonarius* producing OTA were used for comparison.

### Experimental design: optimizing xylanase production

The strategy used in the present study was composed of two Plackett–Burman designs (PB16 and PB12) and one factorial fractional (2^4−1^). As a preliminary study, the source and concentration of carbon and nitrogen were varied to determine which combination provided the highest xylanase yield. A total of ten independent variables were used (PB16): the number of inoculum, pH, salinity (artificial seawater), and concentrations of (NH_4_)_2_SO_4_, peptone, sucrose, xylan and agro industrial residues (sugar cane bagasse, wheat bran, and rice straw). Each component was added in different combinations according to Table 2. The variables’ agitation and temperature were fixed at 140 rpm and 28 °C, respectively. The artificial seawater (ASW) was composed of (g/L of distilled water): 23.93 NaCl, 4.0 Na_2_SO_4_, 0.68 KCl, 0.19 NaHCO_3_, 0.098 KBr, 0.026 H_3_BO_3_, 0.003 NaF, 10.83 MgCl_2_·6H2O, 1.51 CaCl·2H_2_O, and 0.02 SrCl_2_·6H_2_O (Kester et al. 1967). For each design, 4 replicates were used at the central point.

In the second design (PB12) the effect of five independent variables were studied: pH, peptone, wheat bran, xylan, and the number of inocula according to data presented in Table 3. The variables’ agitation and temperature was fixed at 140 rpm and 28 °C, respectively and the variable bagasse was set at 60 g/L.

The factorial fractional design 2^4−1^ was composed of 12 runs and 4 independent variables: pH, peptone, the amount of inoculum, and rice straw (Table 4). The variables agitation and temperature was fixed at 140 rpm and 28 °C, respectively and the variable bagasse was set at 60 g/L.

The results were analyzed using the software STATISTICA 7. A significance level of 10 % (p > 0.1) was considered for the variables used in the present study.

### Protein determination

Protein concentration was determined by the method reported by Lowry et al. (1951) using bovine serum albumin as standard.

### Crude enzyme characterization: influence of pH and temperature, and thermal stability

Culture filtrates from optimized conditions were used to study the properties of xylanase. The effect of pH on enzyme activities (at 40 °C) was determined using different reaction buffers: 0.1 mol/L McIlvaine (pH 3.0–8.0); 0.1 mol/L sodium acetate (pH 4.0–5.6); and 0.1 mol/L Tris–HCl (pH 8.0–9.0). The temperature profiles for the activities of xylanases were obtained by determining the activities at different reaction temperatures (30, 35, 40, 45, 50, 55, 60, 65, and 70 °C) in 0.1 mol/L McIlvaine buffer (pH 5.0). To determine the pH stability, the culture filtrates were diluted in each of the different buffers and incubated at 4 °C for 24 h. To evaluate the thermal stability, the culture filtrates were incubated for 1 h at the temperatures cited above.

## Results

Among the 493 marine-derived fungi studied, 112 were able to degrade xylan in the solid medium under the conditions applied in the present work. The enzymatic activity ranged from 0.25 to 49.41 U/mL and 31 fungi produced more than 10 U/mL of the studied enzyme (Table 1). The largest number of fungi able to produce xylanase was recovered from marine sponges (Table 1).

**Table 1:** Xylanase production by marine-derived fungi (the top 10) after 7 days of cultivation at 28 °C and 140 rpm

Fungus code	Marine source	Xylanase (U/mL)
LAMAI 31	*D. reticulatum*	49.41
LAMAI 155	*D. reticulatum*	30.65
LAMAI 67	*D. reticulatum*	25.20
LAMAI 452	*D. reticulatum*	23.39
LAMAI 74	*D. reticulatum*	21.48
LAMAI 12	*Didemnum* sp.	20.58
LAMAI 288	*D. reticulatum*	19.22
LAMAI 414	*Petromica citrina*	19.06
LAMAI 694	Sponge	18.70
LAMAI 684	Sponge	17.69

**Fig. 1:**
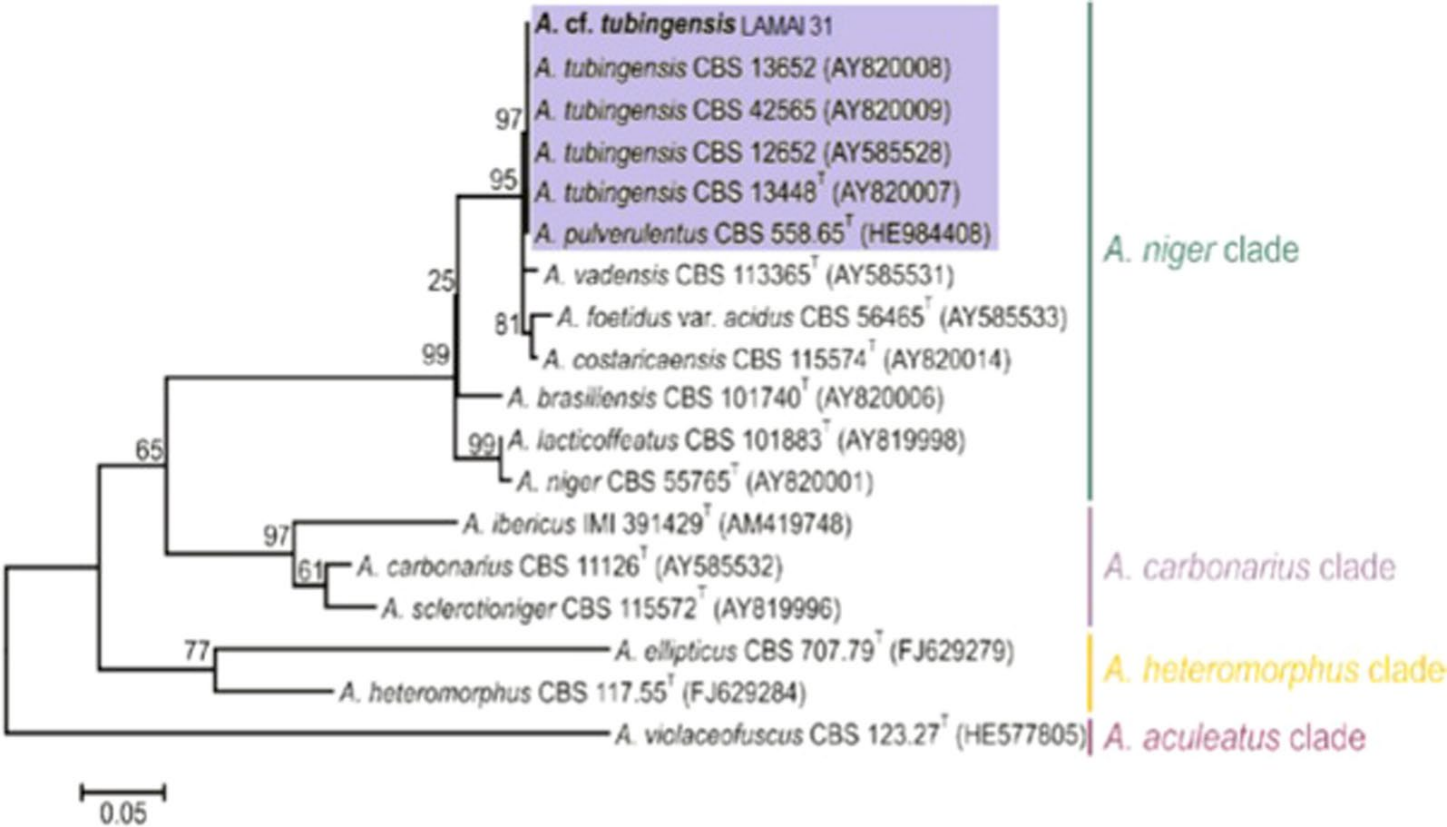
Phylogenetic affiliation of the isolate LAMAI 31 based on the alignment of 18 β-tubulin gene sequences. The analysis was conducted under the maximum-likelihood algorithm. Numbers on branches indicate *bootstrap* support (only values above 50 % are shown). Main clades are named following Jurjevic et al. (2012). The voucher accession numbers in culture collections follow the taxon names and GenBank accessions are given in parenthesis. ^T^ex-type culture

Strain LAMAI 31 had the best result of xylanase production and was submitted to molecular taxonomic characterization. The alignment and comparison of LAMAI 31 β-tubulin sequence showed high similarity with two different species: *Aspergillus* cf. *tubingensis* and *Aspergillus pulverulentus*. Phylogenetic analysis using 17 representative sequences obtained from Jurjevic et al. (2012) (Fig. 1) identified the fungus LAMAI 31 as *Aspergillus* cf*. tubingensis* (*A. niger* clade).

Marine-derived fungus *A.* cf*. tubingensis* LAMAI 31 was subjected to toxin production assays. According to Visconti et al. (2001) the retention time of fumonisin B_2_ standard was 15 min. Based on this methodology, there was no production of fumonisin B_2_ by the fungus *A. cf. tubingensis* LAMAI 31. Additionally, using the Filtenborg et al. (1983) agar plug technique, no ochratoxin A was detected in the experiments with fungus *A.* cf. *tubingensis* LAMAI 31.

Because *A*. cf. *tubingensis* LAMAI 31 is not a toxin producer, it was selected and submitted to the experiments related to the influence of different factors on the enzymatic production aiming for the optimization of the culture conditions. After the conduction of the first PB design (PB 16 with 10 variables), enzymatic activity reached 501.9 U/mL (run 12) in a medium composed of peptone (20 g/L), sugar cane bagasse (60 g/L), rice straw (60 g/L), xylan (20 g/L), 3 pellets of inoculum, and pH 7 (Table 2).

**Table 2:** Plackett–Burman experimental design matrix PB16 (real and coded values) with their respective responses (enzymatic activity)

Run	pH	ASW (%)	Variables^a,b^							Inoculum (pellet)	Xylanase (U/mL)
			(NH_4_)_2_SO_4_	Peptone	SB	WB	RS	Sucrose	Xilan		
1	7 (+1)	0 (−1)	0 (−1)	0 (−1)	60 (+1)	0 (−1)	0 (−1)	20 (+1)	0 (−1)	3 (+1)	16.1
2	7 (+1)	100 (+1)	0 (−1)	0 (−1)	0 (−1)	60 (+1)	0 (−1)	0 (−1)	20 (+1)	3 (+1)	45.1
3	7 (+1)	100 (+1)	4 (+1)	0 (−1)	0 (−1)	0 (−1)	60 (+1)	0 (−1)	20 (+1)	1 (−1)	1.8
4	7 (+1)	100 (+1)	4 (+1)	20 (+1)	0 (−1)	0 (−1)	0 (−1)	20 (+1)	0 (−1)	1 (−1)	2.4
5	5 (−1)	100 (+1)	4 (+1)	20 (+1)	60 (+1)	0 (−1)	0 (−1)	0 (−1)	0 (−1)	3 (+1)	0
6	7 +1)	0 (−1)	4 (+1)	20 (+1)	60 (+1)	60 (+1)	0 (−1)	0 (−1)	20 (+1)	1 (−1)	494.4
7	5(−1)	100 (+1)	0 (−1)	20 (+1)	60 (+1)	60 (+1)	60 (+1)	0 (−1)	0 (−1)	1 (−1)	109.3
8	7 (+1)	0 (−1)	4 (+1)	0 (−1)	60 (+1)	60 (+1)	60 (+1)	20 (+1)	0 (−1)	1 (−1)	290.5
9	7 (+1)	100 (+1)	0 (−1)	20 (+1)	0 (−1)	60 (+1)	60 (+1)	20 (+1)	0 (−1)	3 (+1)	38.3
10	5 (−1)	100 (+1)	4 (+1)	0 (−1)	60 (+1)	0 (−1)	60 (+1)	20 (+1)	20 (+1)	3 (+1)	15.3
11	5 (−1)	0 (−1)	4 (+1)	20 (+1)	0 (−1)	60 (+1)	0 (−1)	20 (+1)	20 (+1)	3 (+1)	83.1
12	7 (+1)	0 (−1)	0 (−1)	20 (+1)	60 (+1)	0 (−1)	60 (+1)	0 (−1)	20 (+1)	3 (+1)	501.9
13	5 (−1)	100 (+1)	0 (−1)	0 (−1)	60 (+1)	60 (+1)	0 (−1)	20 (+1)	20 (+1)	1 (−1)	51.3
14	5 (−1)	0 (−1)	4 (+1)	0 (−1)	0 (−1)	60 (+1)	60 (+1)	0 (−1)	0 (−1)	3 (+1)	50.3
15	5 (−1)	0 (−1)	0 (−1)	20 (+1)	0 (−1)	0 (−1)	60 (+1)	20 (+1)	20 (+1)	1 (−1)	86.5
16	5 (−1)	0 (−1)	0 (−1)	0 (−1)	0 (−1)	0 (−1)	0 (−1)	0 (−1)	0 (−1)	1 (−1)	0
17 (C)	6 (0)	50 (0)	2 (0)	10 (0)	30 (0)	30 (0)	30 (0)	10 (0)	10 (0)	2 (0)	370.2
18 (C)	6 (0)	50 (0)	2 (0)	10 (0)	30 (0)	30 (0)	30 (0)	10 (0)	10 (0)	2 (0)	246.7
19 (C)	6 (0)	50 (0)	2 (0)	10 (0)	30 (0)	30 (0)	30 (0)	10 (0)	10 (0)	2 (0)	319.8
20 (C)	6 (0)	50 (0)	2 (0)	10 (0)	30 (0)	30 (0)	30 (0)	10 (0)	10 (0)	2 (0)	381.0

The experiments were conducted at 28 °C and 140 rpm

*c* central point, *SB* sugarcane bagasse, *WB* wheat bran, *RS* rice straw

^a^ In parenthesis coded values

^b^ Plackett–Burman values in g/L

According to statistical analysis two variables were considered significant, since they showed a p value less than 0.1 (p < 0.1): ASW with negative effect and sugarcane bagasse with positive effect (Additional file 1: Table S1). Therefore, the variable salinity was discarded and not considered in the next PB design. In contrast, the variable sugarcane bagasse was maintained and fixed in 60 g/L.

In the second PB (PB12 with five variables) the best result of enzymatic production was 629.6 U/mL (run 2) (Table 3), which was composed of peptone (40 g/L), rice straw (90 g/L) pH 6, and three pellets of inoculum. In this experiment, the variables’ peptone and the amount of inoculum were significant (p < 0.1) and showed a positive effect on the enzymatic production (Additional file 1: Table S2).

**Table 3:** Plackett–Burman experimental design matrix PB12 (real and coded values) with their respective responses (enzymatic activity)

Run	Variables^a,b^			Inoculum (pellets)	pH	Xylanase (U/mL)
	Peptone	RS	Xylan			
1	40 (1)	30 (−1)	40 (1)	3 (−1)	6 (−1)	104.48
2	40 (1)	90 (1)	0 (−1)	5 (1)	6 (−1)	629.67
3	0 (−1)	90 (1)	40 (1)	3 (−1)	8 (1)	78.36
4	40 (1)	30 (−1)	40 (1)	5 (1)	6 (−1)	496.26
5	40 (1)	90 (1)	0 (−1)	5 (1)	8 (1)	609.78
6	40 (1)	90 (1)	40 (1)	3 (−1)	8 (1)	180.24
7	0 (−1)	90 (1)	40 (1)	5 (1)	6 (−1)	71.27
8	0 (−1)	30 (−1)	40 (1)	5 (1)	8 (1)	18.58
9	0 (−1)	30 (−1)	0 (−1)	5 (1)	8 (1)	26.64
10	40 (1)	30 (−1)	0 (−1)	3 (−1)	8 (1)	93.66
11	0 (−1)	90 (1)	0 (−1)	3 (−1)	6 (−1)	129.67
12	0 (−1)	30 (−1)	0 (−1)	3 (−1)	6 (−1)	139.75
13 (C)	20 (0)	60 (0)	20 (0)	4 (0)	7 (0)	358.53
14 (C)	20 (0)	60 (0)	20 (0)	4 (0)	7 (0)	421.82
15 (C)	20 (0)	60 (0)	20 (0)	4 (0)	7 (0)	439.13
16 (C)	20 (0)	60 (0)	20 (0)	4 (0)	7 (0)	439.43

The experiments were conducted at 28 °C and 140 rpm

*c* central point, *RS* rice straw

^a^ In parenthesis coded values

^b^ Values in g/L

Considering the results obtained in PB 12, a Factorial fractional design (FF) was structured, with the four variables that had a positive effect in the last PB, totalizing 12 experiments (Table 4). The results showed that FF design was not effective, since the enzymatic activity values were much lower than the previous obtained in PB16 and PB 12. The best result was achieved in run 1 (135.30 U/mL), with the following culture conditions: pH 6, five pellets of inoculum, rice straw (70 g/L), and in the absence of peptone. The second best result was obtained in run 4 (124.37 U/mL), which differed from run 1 in pH (8) and amount of inoculum (seven pellets) (Table 4).

**Table 4:** Factorial fractional design matrix FF 2^4−1^ (real and coded values) with their respective responses (enzymatic activity). The experiments were conducted at 28 °C and 140 rpm

Run	Variables^a.b^				Xylanase (U/mL)
	pH	Inoculum (pellets)	Peptone	RS	
1	6 (−1)	5 (−1)	0 (−1)	70 (−1)	135.30
2	8 (1)	5 (−1)	0 (−1)	110 (1)	91.21
3	6 (−1)	7 (1)	0 (−1)	110 (1)	104.78
4	8 (1)	7 (1)	0 (−1)	70 (−1)	124.37
5	6 (−1)	5 (−1)	80 (1)	110 (1)	63.69
6	8 (1)	5 (−1)	80 (1)	70 (−1)	61.43
7	6 (−1)	7 (1)	80 (1)	70 (−1)	79.90
8	8 (1)	7 (1)	80 (1)	110 (1)	42.97
9 (C)	7 (0)	6 (0)	40 (0)	90 (0)	73.12
10 (C)	7 (0)	6 (0)	40 (0)	90 (0)	72.74
11 (C)	7 (0)	6 (0)	40 (0)	90 (0)	88.19
12 (C)	7 (0)	6 (0)	40 (0)	90 (0)	50.50

*c* central point, *RS* rice straw

^a^ Coded values in parenthesis

^b^ Values in g/L

In this experiment, the variables peptone and rice straw were statistically significant, but had a negative effect. The only variable with a positive effect was the amount of inoculum.

Considering the results obtained in the three experimental designs (two PB and one FF), the optimized condition was considered the one applied in the run 2 of PB12, which showed 629.67 U/mL of enzymatic activity. To confirm this result a validation assay was carried out and the enzymatic activity was measured during 168 h of incubation in the optimized medium (40 g/L peptone, 90 g/L rice straw, five pellets of inoculum, pH 6 and 60 g/L sugarcane bagasse). Confirmatory experiments were conducted in triplicate, during 7 days of incubation at 140 rpm and 28 °C.

**Fig. 2:**
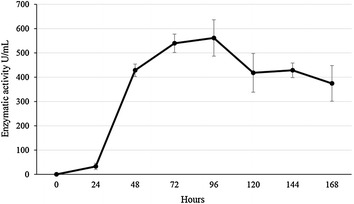
Xylanase activities of *A.* cf. *tubingensis* LAMAI 31 during 168 h incubation under validated conditions (run 2, PB16)

After the first 24 h it was possible to identify the presence of the enzyme (32.65 U/mL) in the culture filtrate of *A.* cf. *tubingensis* LAMAI 31. The peak of enzymatic production was observed after 96 h of cultivation (561.59 U/mL), following by a decrease in the activity, which reached 374.47 U/mL after 168 h of incubation (Fig. 2).

Although the best activity was on the 4th day (96 h) of the validation assay, the amount of protein varied during the period studied (Additional file 1: Table S3). The highest value of protein in the 1st days may be related to proteins already present in the culture medium. After 96 h of incubation, the fungus *A.* cf*. tubingensis* LAMAI 31, had a higher enzymatic activity (561.59 U/mL), however, the specific activity (197.02 mg/mL) was lower after 72 h of incubation (209.26 mg/mL).

**Fig. 3:**
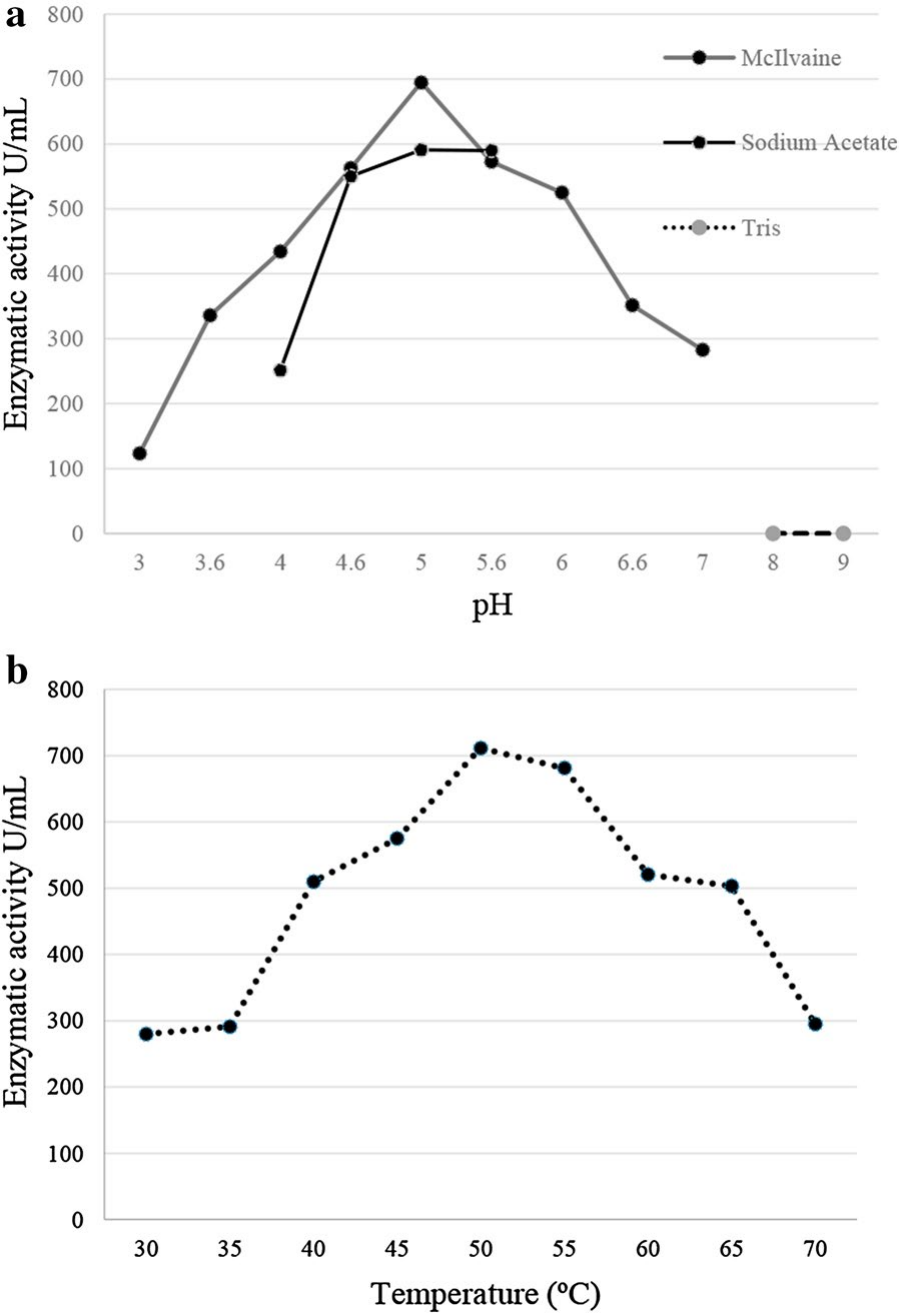
Optimum pH **a** and temperature **b** for xylanase activity. **a** The reactions were performed at 40 °C in buffers: 0.1 McIlvaine mol/L, sodium acetate 0.1 mol/L, and Tris–HCl 0.1 mol/L, **b** The reactions were performed using McIlvaine 0.1 mol/L. Standard deviation was <0.01

Xylanase produced by the marine-derived fungus *A.* cf. *tubingesis* LAMAI 31 during validation assay showed activity in both acidic and alkaline pH. The activity of the crude enzyme was observed in the range of pH 3.0–7.0 (Fig. 3a). The optimal enzymatic activity was at pH 5.0 (694.64 U/mL) in McIIvaine buffer, after 4 days of cultivation. Lower activity (123.7 U/mL) was observed at pH 3. There was no enzymatic activity at pH above 7.0.

The enzyme showed activity at all temperatures studied. However, optimum activity was at 50 °C (711.43 U/mL) (Fig. 3b). Xylanase activity was gradually increasing from the lowest temperature up to 50 °C. From 55 °C enzymatic activity was reduced until reaching the value of 294.98 U/mL at 70 °C.

**Fig. 4:**
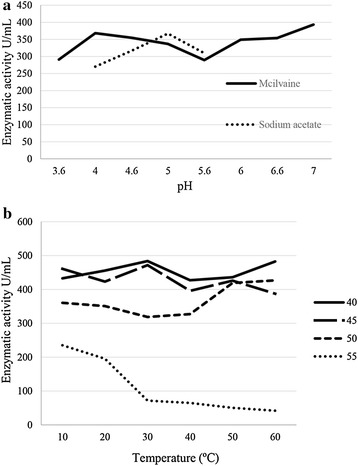
Stability of xylanase activity under different pH (**a**) and temperatures (**b**). **a** Samples were incubated for 24 h at 40 °C in the absence of substrate and with buffers: McIlvaine 0.1 mol/L, sodium acetate and 0.1 mol/L; **b** Samples were incubated at 40, 45, 50 and 55 °C in the absence of substrate. Standard deviation was <0.01

The stability of the enzyme was evaluated at different pH and temperatures. The best result was obtained at pH 7.0 after 24 h of incubation (Fig. 4a). The buffer McIlvaine (pH 4.0 and 4.6) showed an increase in the stability of the enzyme, while sodium acetate buffer showed the greatest enzymatic stability at pH 5.0 and 5.6.

Concerning temperature stability, the enzyme showed good stability at 40 °C and 45 °C, maintaining the activity during 60 min of incubation (Fig. 4b). At 40 °C and 50 °C the enzyme remained stable with an increase in activity after 50 min of incubation. The enzyme was not stable at 55 °C and after 10 min of incubation its activity was low, decreasing gradually after 30 min of incubation.

## Discussion

In the present study, marine-derived fungi that showed more expressive xylanase activities were isolated from marine sponges, particularly *Dragmacidon reticulatum*. Interactions between sponges and microorganisms occur in many forms, where the microbes can represent: (1) food sources (Reiswig 1975), (2) pathogens/parasites (Bavestrello et al. 2000; Hummel et al. 1988; Webster et al. 2002), or (3) mutualistic symbionts (Wilkinson 1980). According to Wang et al. (2006) considering the fact that the sponges capture food by filtration (e.g. phytoplankton, algae, plankton, and other organisms), the microorganisms that live in sponges must be able to produce enzymes capable to convert in organic matter.

The strain LAMAI 31 recovered from *D. reticulatum*, identified as *A.* cf*. tubingensis*, showed the best result of xylanase production and is considered a facultative marine fungus, since other representatives of this species have been found in several terrestrial environments. Fungi from genus *Aspergillus* are salt-tolerant and were reported as natural inhabitants of marine invertebrates (Menezes et al. 2010).

*Aspergillus* section *Nigri* have been considered as a source of Ochratoxin A (OTA) and fumonisins in many food and crops (Iamanaka et al. 2005; Copetti et al. 2010). OTA is a nephrotoxic mycotoxin classified by the International Agency for Research on Cancer (IARC and Ochratoxin 1993) as group 2B, a possible carcinogenic agent for humans. In our study, Ochratoxin A and fumonisis B_2_ were not detected in *A.* cf*. tubingensis* LAMAI 31, highlighting the potential of this fungus for being used in industrial processes.

Culture conditions clearly influence the production of enzymes and the best way to improve their production is to apply experimental design and statistical analysis (Levin et al. 2008; Bonugli-Santos et al. 2010). In the present study, the application of this approach resulted in an increase of 12.7 times in the enzymatic activity of *A.* cf. *tubingensis* LAMAI 31, which ranged from 49.41 U/mL to 629.6 U/mL. In a previous study reported by Raghukumar et al (2004), a representative of marine derived-fungus from the genus *Aspergillus* (*A. niger* NIOCC 3 isolated from mangrove leaf detritus) was able to produce xylanase (54 U/L). Results from the present work revealed the importance of experimental design to the evaluation of the main factors that affect the enzymatic production in order to optimize culture conditions.

The xylanase produced by *A.* cf. *tubingensis* LAMAI 31 was favored in the presence of sugarcane bagasse and was inhibited in the presence of artificial seawater. Other studies have shown that xylanase was induced by sugarcane bagasse (Raghukumar et al. 2004; Maciel et al. 2008). The better conditions for xylanase production by *A.* cf. *tubingensis* LAMAI 31 was in the presence of peptone (40 g/L), rice straw (90 g/L), sugarcane bagasse (60 g/L), pH 6, and three pellets of inoculum. Peptone as the nitrogen source has influence in the growth of the fungus and in the induction of the enzyme. According to Ho (2014) different sources of nitrogen (organic and inorganic) can promote impact on the capacity of growth and enzymatic production.

The peak of xylanase produced by *A.* cf. *tubingensis* LAMAI 31 in the validation assay was in 96 h of cultivation. However, the highest specific activity was in 72 h of cultivation. After 96 h, enzymatic production showed a decrease, probably as a result of nutrient depletion or due to the action of proteases. These results differ from those obtained by Mostafa et al. (2014), where production of xylanase by a consortium of microorganisms (*Aspergillus flavus, Cladosporium sphaerospermum*, and *Epicoccum purpurascens*) increased after 96 h of cultivation.

Analyses of the crude enzymatic broth showed peak activity at pH 5.0 (694.64 U/mL) and 50 °C (711.43 U/mL). According to Polizeli et al. (2005) the majority of *Aspergillus* species have optimal pH and temperature in the range of 4.0–6.0 and 40–80 °C, respectively. Additionally, data from previous studies show that the temperature and pH optima of purified enzymes produced by marine-derived fungal strains ranged from 35–75 °C and 3.0–11.0, respectively (Bonugli-Santos et al. 2015). These data corroborate the results obtained in the present study (crude broth), where the highest xylanase activities occurred from 40 to 65 °C and from pH 4.6 to 5.6. The only study found in the consulted literature related to production of xylanase by an *Aspergillus* specie from marine origin (*A. niger* NIOCC 3) showed that the xylanase produced by this fungus had the temperature and pH optimum at 50 °C and 3.5, respectively (Raghukumar et al. 2004).

Xylanase produced by *A.* cf. *tubingensis* LAMAI 31 was stable at temperatures 40, 45 and 50 °C (after 1 h of cultivation) and showed great stability at pH 7.0 (after 24 h of cultivation). High stability and the maintenance of the activity level over a long period are two important points to be considered for the enzymatic application (Kapoor et al. 2008).

The absence of pathogenicity and the ability to produce great amounts of a stable xylanase active in both acidic and alkaline pH, with highest specific activity in 72 h of incubation, emphasize the importance of *A.* cf. *tubingensis* LAMAI 31 as a marine genetic resource for biotechnological applications. These results stimulate new studies related to enzymatic purification, the definition of kinetic parameters, and molecular characterization (biological engineering) in order to increase the knowledge of xylanase from marine origin and also to compare it with terrestrial xylanases produced by representatives of the genus *Aspergillus* from section *Nigri*.

## Additional file

10.1186/s13568-016-0194-z **Table S1.** Main effects and statistical significance (PB16). **Table S2.** Main effects and statistical significance (PB12). **Table S3.** Specific activity of the crude enzyme under optimized conditions.
