# Supplementary material for: Marine-derived fungus Aspergillus cf. tubingensis LAMAI 31: a new genetic resource for xylanase production
Source: AMB Express. 2016 Mar 24;6:25. doi: 10.1186/s13568-016-0194-z (PMC4805677; doi:10.1186/s13568-016-0194-z)
Supplement: Supplementary file 1 — 10.1186/s13568-016-0194-z Table S1. Main effects and statistical significance (PB16). Table S2. Main effects and statistical significance (PB12). Table S3. Specific activity of the crude enzyme under optimized conditions. [file 13568_2016_194_MOESM1_ESM.docx]

**AMB Express**

**Marine-derived fungus *Aspergillus* cf. *tubingensis* LAMAI 31: a new genetic resource for xylanase production**

Juliana A. dos Santos^1^, Juliana M.F. Vieira^1^, Alexandre Videira^1^, Lucas A. Meirelles^1^, André Rodrigues^1^, Marta H.Taniwaki^2^, Lara D. Sette^1*^

^1^Departamento de Bioquímica e Microbiologia, Universidade Estadual Paulista Júlio de Mesquita Filho - UNESP, Rio Claro, SP, Brazil.

^2^Instituto de Tecnologia de Alimentos - ITAL, Campinas, SP, Brazil.

* Corresponding author at: Departamento de Bioquímica e Microbiologia - IB, Universidade Estadual Paulista Júlio de Mesquita Filho - UNESP, 24A, 1515 - 13506-900, Rio Claro, SP, Brazil. Tel: +55 19 3526-4171.

E-mail address: larasette@rc.unesp.br (L.R. Sette)

**Table S1. Main effects and statistical significance (PB16)**

| Variables | Effect | P-value |
| --- | --- | --- |
| pH | 124.332 | 0.124019 |
| ASW | -157.427 | 0.060222* |
| (NH_4_)_2_SO_4_ | 11.143 | 0.882498 |
| peptone | 105.698 | 0.183107 |
| cane bagasse | 146.423 | 0.076804* |
| wheat bran | 67.285 | 0.382503 |
| rice straw | 50.166 | 0.510889 |
| sucrose | -77.388 | 0.318504 |
| inoculum | -35.758 | 0.637270 |
| xilan | 96.582 | 0.220102 |

*significant values (p<0.1)

**Table S2. Main effects and statistical significance (PB12)**

| Variables | Effect | P-value |
| --- | --- | --- |
| Peptone | 274.969 | 0.010767* |
| Rice straw | 136.601 | 0.151502 |
| Xilan | -113.328 | 0.226647 |
| Inoculum | 187.674 | 0.058677* |
| pH | -93.974 | 0.310499 |

* significant values (p<0.1)

**Table S3.** Specific activity of the crude enzyme under optimized conditions

| Time (h) | Enzymatic Activity (U/mL) | Total Protein (mg/mL) | Specific Activity  (mg/mL) |
| --- | --- | --- | --- |
| 24 | 32.65 | 4.11 | 7.95 |
| 48 | 428.51 | 3.02 | 142.03 |
| 72 | 539.76 | 2.58 | 209.26 |
| 96 | 561.59 | 2.85 | 197.02 |
| 120 | 418.13 | 2.78 | 150.18 |
| 144 | 428.57 | 3.27 | 131.15 |
| 168 | 374.47 | 2.71 | 138.29 |
